# CD22 Blockage Restores Age-Related Impairments of Microglia Surveillance Capacity

**DOI:** 10.3389/fimmu.2021.684430

**Published:** 2021-06-01

**Authors:** Vanessa Aires, Claire Coulon-Bainier, Anto Pavlovic, Martin Ebeling, Roland Schmucki, Christophe Schweitzer, Erich Kueng, Simon Gutbier, Eva Harde

**Affiliations:** ^1^ Roche Pharma Research and Early Development, Neuroscience and Rare Diseases Discovery and Translational Area, Roche Innovation Center Basel, F. Hoffmann-La Roche Ltd, Basel, Switzerland; ^2^ Department of Neurology, Medical Center – University of Freiburg, Freiburg, Germany; ^3^ Faculty of Biology, University of Freiburg, Freiburg, Germany; ^4^ Roche Pharma Research and Early Development, Pharmaceutical Sciences, Roche Innovation Center Basel, F. Hoffmann-La Roche Ltd, Basel, Switzerland; ^5^ Roche Pharma Research and Early Development, Therapeutic Modalities, Roche Innovation Center Basel, F. Hoffmann-La Roche Ltd, Basel, Switzerland

**Keywords:** CD22, microglia, two-photon imaging, surveillance, iPSC macrophages, phagocytosis, aging, AD (Alzheimer’s disease)

## Abstract

Microglia, the innate immune cells of the brain, are essential for maintaining homeostasis by their ramified, highly motile processes and for orchestrating the immune response to pathological stimuli. They are implicated in several neurodegenerative diseases like Alzheimer’s and Parkinson’s disease. One commonality of these diseases is their strong correlation with aging as the highest risk factor and studying age-related alterations in microglia physiology and associated signaling mechanism is indispensable for a better understanding of age-related pathomechanisms. CD22 has been identified as a modifier of microglia phagocytosis in a recent study, but not much is known about the function of CD22 in microglia. Here we show that CD22 surface levels are upregulated in aged versus adult microglia. Furthermore, in the amyloid mouse model PS2APP, Aβ-containing microglia also exhibit increased CD22 signal. To assess the impact of CD22 blockage on microglia morphology and dynamics, we have established a protocol to image microglia process motility in acutely prepared brain slices from CX3CR1-GFP reporter mice. We observed a significant reduction of microglial ramification and surveillance capacity in brain slices from aged versus adult mice. The age-related decrease in surveillance can be restored by antibody-mediated CD22 blockage in aged mice, whereas surveillance in adult mice is not affected by CD22 inhibition. Moreover to complement the results obtained in mice, we show that human iPSC-derived macrophages exhibit an increased phagocytic capacity upon CD22 blockage. Downstream analysis of antibody-mediated CD22 inhibition revealed an influence on BMP and TGFβ associated gene networks. Our results demonstrate CD22 as a broad age-associated modulator of microglia functionality with potential implications for neurodegenerative disorders.

## Introduction

Microglia are the resident immune cells of the brain and are manifold involved in shaping their surrounding tissue from development (1–3) till aging (4–6). Besides their role in pathogen recognition and immune response orchestration, microglia are also vital under homeostatic conditions. They contribute substantially to maintaining homeostasis in the brain, by removing cellular debris, aggregated proteins and apoptotic cells (2, 7). With their ramified and highly motile processes, microglia constantly monitor the brain parenchyma to sense and counteract disturbances in the central nervous system (8). During aging however, microglia undergo phenotypic changes, characterized by the reduced expression of cytoskeleton-regulating genes (9) consistent with the impaired surveillance and reduced lesion response of microglia (10). Moreover, during aging microglia loose their homeostatic signature (11) and adopt a pro-inflammatory state, accompanied by increased expression of pro-inflammatory cytokines (12), reduced phagocytic activity and the accumulation of insoluble cargo (13).

Aging is one of the biggest risk factors for neurodegenerative disorders (14) and age-related disturbances in microglia functionality could contribute to the initiation and progression of various neurodegenerative diseases. Therefore, understanding the effect of aging on microglial behavior and whether age-related dysfunctionality can be modulated is crucial to comprehend the ambiguous role of microglia in age-related neurodegenerative diseases. Ultimately, new findings in this area will be essential for the identification of new microglia-specific targets in drug discovery.

A recent study in murine cells identified CD22 as an age-associated modifier of microglial phagocytosis using a CRISPR-Cas9 knockout screen (15). *In vivo* antibody-mediated blockage of CD22 led to the restoration of phagocytosis, reprogrammed microglia toward a homeostatic transcriptional state and ultimately improved the cognitive functions of aged mice (15). CD22, a sialic-acid-binding immunoglobulin-like lectin (SIGLEC), is usually expressed on B-cells where it functions as an inhibitory co-receptor of the B-cell receptor (16). Besides its involvement in phagocytosis in murine phagocytosis, the function of CD22 in microglia is largely unknown.

Here, we report that CD22 is upregulated by microglia during aging and in plaque-associated microglia in the amyloid mouse model PS2APP. Investigating microglia can be challenging, since they are highly sensitive to changes in their environment and rapidly loose key characteristics upon isolation (17, 18). Therefore, it is essential to study microglia in their native tissue environment. Using two-photon live microscopy of acute brain slices prepared from CX3CR1-GFP reporter animals in combination with a nearly fully automated analysis pipeline, we show that microglial dynamic behavior can be reliably assessed by this technology. We demonstrate that surveillance is strongly reduced in aged versus adult microglia. Interestingly, antibody-mediated blockage of CD22 in brain slices of aged mice restores the age-related microglial hypo-ramification and the reduced surveillance capacity. To connect these murine based findings to human biology, we addressed the impact of CD22 blockage on human iPSC-derived macrophages by studying effects on phagocytosis and transcriptional regulation. Interestingly, in human iPSC macrophages inhibition of CD22 promoted the phagocytosis of Aβ-coated beads and modulated key regulatory networks at the gene expression level. By using a joint approach of murine and human model systems, we show that CD22 blockage acts as a broad age-related modifier of microglia functions.

## Materials and Methods

### Animals

All animal experiments were performed with the permission of the Swiss Cantonal Veterinary Office. The mice used in this study were bred and maintained in temperature and humidity controlled facilities. Food and water was available *ad libitum*. The following lines were used in the study: CX3CR1-GFP (19) and a cross of CX3CR1-GFP to PS2APP (20). Both transgenes were kept in a heterozygous condition. To minimize gender-dependent heterogeneity of microglia phenotype (21, 22), only male mice were used in the study. CX3CR1-GFP animals were used at 6-8 months as adult mice and at 13-16 months as aged mice. PS2APP CX3CR1-GFP were around 10 months old.

### Acute Slice Preparation

Animals were anesthetized in an isoflurane chamber and then sacrificed by decapitation. The brain was quickly removed and put in ice-cold NMDG-based artificial cerebral fluid solution (NMDG- ACSF) consisting of the following reagents: 110 mM NMDG, 3 mM KCl, 1.1 mM NaH_2_PO_4_, 25 mM NaHCO_3_, 25 mM D-Glucose, 10 mM L-Ascorbic acid, 3 mM Pyruvic acid, 0.5 mM CaCl_2,_ 10 mM MgCl_2_, 103.02 mM HCl; pH 7.3, 305-310 mOsm. Acute coronal hippocampal slices (300 μm thick) of adult (7-8 months) or aged (13-16 months) mice were sliced using a vibratome (VT1200, Leica). Dissection and slicing was performed in ice-cold NMDG-ACSF, which was constantly saturated with carbogen (95% O_2_, 5% CO_2_). After slicing and recovery in 37°C warm NMDG-ACSF for 15 min, acute slices were transferred to carbogenated ACSF (at room temperature), containing the following: 119 mM NaCl, 2.5 mM KCl, 2.5 mM CaCl_2_, 1.3 mM MgCl_2_, 1 mM NaH_2_PO_4_, 11 mM Glucose, 26.2 mM NaHCO_3_; 290 mOsm. Slices were kept in ACSF until imaged, but stayed at least 30 min in ACSF for acclimatization.

For antibody treatment, ACSF acclimatization is followed by transfer to incubation chambers, containing carbogenated ACSF with either CD22 antibody (BioXcell, BE0011; 5 μg/ml) or IgG isotype control (BioXcell, BE0083; 5 μg/ml). Sections stayed in incubation chambers for at least 90 minutes.

### Cranial Window Surgery

The animal was anaesthetized using a triple shot standard injection protocol consisting of a combination of Fentanyl (0.05 mg/kg), Medetomidine (0.5 mg/kg) and Midazolam (5 mg/kg) in a subcutaneous injection and the depth of anesthesia was regularly assessed through toe pinching. Body temperature was maintained at 36.5–37.5°C using a heating pad connected to a controller, and the eyes were covered with eye protecting gel. During experimentation, heart rate (mouse = 300–450 beat/min) and O_2_ saturation (SpO2; mouse >95%) were monitored using a pulse oximeter. The head was shaved on the surgical area and cleaned with Betadine, then firmly fixed with ear bars in a stereotaxic frame. The surgical area was locally numbed by s.c. injection of local anesthesia (bupivacaine).

An incision was carefully made over the skin. The periosteum was removed by gently scraping the skull with a scalpel blade and scratches were made on the skull for cement adherence. The skull was then degreased with H_2_O_2_ by cotton swabbing it and dried. All exposed skull bone was covered with 2 layers of I-bond (Kulzer I-Bond total etch) except for the area of the future cranial window and dried with an UV lamp. The area to drill was marked with a 3mm biopsy punch on the left parietal region. Tetric evoflow cement was applied on all the surface of the exposed skull, except the marked area. A circular thinning of the skull around the marked area for the cranial window was created. Bone crumbles were periodically blown away, the drilling region washed with cold saline to avoid heating and dried before restarting drilling. Thickness of the skull in the central groove was regularly checked by gently pushing on the central area of the cranial bone with a fine probe. When the thinned skull moved upon being lightly touched, it was ready to be removed. A large drop of cold saline was added on the thinned skull and the groove was gently pinched with sharp forceps and lifted away. The groove must be kept parallel to the skull’s surface to avoid direct perforation of the dura mater. The cranial window was filled with a drop of ACSF and the 3mm coverslip covered with ACSF was placed in the hole. Tetric Evoflow cement was then applied all around the coverslip to seal the coverslip to the skull and slightly under it, so it is well maintained. The cement is then dried with an UV lamp. The animal was removed from the ear bars and we used an alignment tool to orient the mouse head so the coverslip is horizontal (laser coming vertically from the top of the animal to the coverslip is reflected on it and the reflection is indicating the angle of the coverslip). Then the metal head plate was placed parallel to the cover slip, fixed with Tetric Evoflow cement and dried with UV light.

### Two-Photon Microscopy

Acute slices were imaged with a two-photon imaging system (Scientifica) equipped with a tunable Ti : Sapphire MaiTai DeepSee laser (Spectra Physics) using an excitation wavelength of 850 nm and the following bandpass filters: 450/50 nm for methoxy-X04 and 525/50 nm for GFP. After placing the acute slice on a cover slip, it was positioned under the 20x objective (XLUMPLFLN; Olympus) where it was constantly perfused with carbogenated ACSF. After identifying the CA1 region, one microglia was selected for time-lapse image acquisition. To avoid activated microglia at the borders of the slice, microglia were exclusively imaged at a depth of >80 µm. With a 6x optical zoom, the cell was centered and the soma was focused. Z-dimensions were set ±30 µm of the soma. Microglia selection was based on unbiased criteria such as clear soma recognition and distinction from neighboring cells. In average 8 stacks were recorded consecutively with the dimensions 129.5 µm*129.5 µm*60 µm (X*Y*Z), a resolution of 512px*512px per plane and a step size of 1 µm. Two images were acquired per plane, which were averaged during image processing. One cell per slice was imaged.

For *in vivo* imaging, the animal was transferred to the two-photon microscope right after surgery on a heating plate. Medetomidine (0.25mg/kg every hour) was injected to prolong the anesthesia. The head was fixed through the implanted head plate to the stage. A drop of water was added on the window and the bright field mode was used to center the window in the 10x objective. The 20x objective (XLUMPLFLN; Olympus) was then used with an excitation wavelength of 850 nm and the 525/50 nm bandpass filter to collect the GFP signal. We recorded cells at least 50 µm deep in a stack going ±30 µm with a 6x optical zoom, in the same way we recorded on slices.

### Image Processing & Analysis

Image processing and analysis (**Figures S1A, B**) was performed using ImageJ by custom-written macros (available upon request). For the time-lapse, the maximum intensity Z-projection for each stack was created followed by the x,y registration using the StackReg plugin (23). After cropping the cell using the temporal color code image overlaying all timepoints, automated image correction steps were applied, including background removal, a 2px median filter, photobleaching correction and contrast enhancement. The resulting binarized images were used for analysis. The relative retraction area is the number of pixels in image t_n_ subtracted with the number of pixels from the consecutive image t_n+1_ relative to the total pixel number of t_n_. The relative retraction reflects the percentage of the cell area, which retracted from t_n_ to t_n+1_. For the extension area, the number of pixels in image t_n+1_ were subtracted with the number of pixels from the image t_n_ relative to the pixel number of t_n_. The relative extension area reflects the percentage of pixels in a new location from t_n_ to t_n+1_. The reported motility index is the sum of the relative retractions and extensions and is used as a readout for the process motility of the microglia. By summing up the absolute retraction and extension area, the surveillance index indicates the surveillance capacity of the microglia and reflects the surveilled area.

### Microglia Activation in the Slice Preparation

At the slice surface, microglia morphology resembles an activated phenotype with rounded cell shape and fine, quickly moving processes (**Video S1**) due to tissue damage induced by the slicing. However, within the slice, homeostatic microglia with fine and highly branched processes are observed (for a scan through the slice see **Video S2**). Compounds of choice can be either applied prior (pre-incubation) or during (wash-in) imaging. To assess if the age of the slice after preparation has an effect on microglia behavior, we have plotted the relative motility, surveillance and area values of untreated slices (from experiment in **Figure 3**) versus the time after slice preparation (**Figures S1C–E**). We did not observe any correlation between our readouts and the age of the slice after preparation. Therefore, these results indicate a stable slice preparation with no aberrant activation of microglia observed over time.

### Tracing of Microglia Processes and Sholl Analysis

To trace microglial processes, the maximum intensity Z-projection from the t_0_ time point of each recorded cell was selected. Using Fiji’s SNT (Simple Neurite Tracer) plugin, processes were traced for subsequent Sholl analysis. With a step size of 1 µm, the number of processes crossing the concentric circles, which were arranged around the soma, were counted. This analysis reflects the ramification of microglia.

### Primary Microglia Isolation

After mice were transcardially perfused with ice-cold PBS, whole brains were isolated to obtain single cell suspensions using the Adults Brain Dissociation Kit, mouse and rat (Miltenyi Biotec, 130-107-677) according to the manufacturer’s protocol. Briefly, the brains were chopped into pieces in cold PBS, transferred into gentleMACS™ C Tubes (Miltenyi Biotec, 130-093-237) and digested using the provided enzymes with the gentleMACS™ Dissociator with Heaters (Miltenyi Biotec, 130-096-427). Tissue lysates were resuspended and filtered through 70μm cell strainers, which were subsequently rinsed with PBS. Samples were centrifuged at 1500 rpm for 10min and the supernatant was decanted. This was followed by gradient centrifugation with 30% Percoll (GE Healthcare Life Sciences, 17-0891-01) in PBS (v/v) (2750 rpm for 30 minutes, at 4°C, no brakes). Myelin was removed with a suction pump and the suspensions were filtered through 70 μm cell strainers and centrifuged at 1500 rpm, 4°C for 10 minutes. The samples were then ready for flow cytometry staining.

### Flow Cytometry

Single cell suspensions were incubated with primary antibodies against CD11b (1:400, clone M1/70, BD Biosciences, 565976), CD45 (1:800, clone 30-F11, BD Biosciences, 563890) or CD45 (1:400, clone 30-F11, BD Biosciences, 565079), CD22 (1:100, clone OX-97, Biolegend, 126112) as well as live cell identification using Zombie NIR™ (1:500, Biolegend, 423106) in PBS for 30 min at 4°C. After washing once with PBS, samples were resuspended in 200 µl PBS and analyzed by flow cytometry with a BD LSRFortessa (BD Biosciences) or a BD FACSymphony flow Cytometer (BD Biosciences). To reduce fluorescent spillover, PMT voltages were adjusted with single-stain controls prepared with VersaComp Antibody Capture Bead Kit (Beckman Coulter, B22804) before sample acquisition. Acquired.fcs files were uploaded in FlowJo software (Tree Star), where artefacts arisen from incorrect compensation-matrix calculation were manually corrected.

### *In Vivo* Amyloid-β Plaque Labeling

18h prior microglia isolation or imaging, PS2APP CX3CR1-GFP mice were injected intraperitoneally with methoxy-X04 (5mg/kg), a blood-brain-barrier penetrating dye, which labels amyloid plaques (24).

### Human iPSC-Derived Macrophages

All work with human iPSC-derived macrophages was performed under the respective Swiss legislation, ethical guidelines, and approval. Production and maintenance of human iPSC-derived macrophages was done according to our recently published adapted protocol from van Wilgenburg et al. (25, 26).

Briefly, iPSC maintenance culture dishes (Corning) were coated with 12.5 µg/ml rhLaminin-521 (BioLamina) prior usage. Cells from the utilized human iPSC cell line Bioneer WT (BIONi010-C; Bioneer) were seeded and cultured in mTesR1 media (StemCell Technologies) at 37°C with 5% CO_2_ with daily media changes. At 90% confluency, cells were passaged by washing the cells once with PBS and detaching with Accutase™ (Innovative Cell Technologies) for 2 to 5 min at 37°. After removal of Accutase™ by centrifugation, cells were either used for maintenance or induction of differentiation. Cell quality was controlled by STR profiling, SNP phenotyping and karyotyping after banking. Sub-culturing was reduced to an absolute minimum to avoid genetic drift.

To initiate macrophage differentiation, embryoid bodies (EBs) were generated as described previously (25). For EB generation, iPSCs were seeded onto AggreWell 800 plates (StemCell Technologies). Per well, 2 ml of a single cell suspension of 4*10^6^ cells in mTesR1, supplemented with 10 mM ROCK inhibitor (Y27632, StemCell Technologies), were added. Centrifugation at 100 g for 3 min assured an even cell distribution. The following day, 75% of the mTeSR1 media were exchanged with mTeSR1 media supplemented with 50 ng/ml rhBMP4 (Biotechne), 50 ng/ml rhVEGF (Biotechne), and 20 ng/ml rhSCF (Biotechne) to start mesoderm and subsequent hemogenic endothelium induction. This medium exchange was repeated for the next 2 days. On day 4 of differentiation, EBs were harvested and transferred to factory medium, consisting of X-VIVO 15 medium (Lonza) supplemented with 2 mM GlutaMAX (Thermo Fisher Scientific), 10 U/ml Penicillin/Streptomycin (Thermo Fisher Scientific), 50 µg/ml Mercaptoethanol (Thermo Fisher Scientific), 100 ng/ml rhMCSF (Miltenyi Biotech), and 25 ng/ml rhIL3 (Miltenyi Biotech). EBs were plated in growth factor reduced Matrigel (Corning) precoated cell culture vessels at a density of 1 EB/cm^2^. Myeloid factories were matured as reported (25).

### *In Vitro* Phagocytosis Assay

iPSC-derived macrophages were plated onto a 96 well plate (Falcon, 353219) at a density of 30000 cells per well. The following compounds were added respectively to assess their impact on phagocytosis: CD22 antibody (BioXcell, BE0011; 5 μg/ml), IgG isotype control (BioXcell, BE0083; 5 μg/ml) and Cytochalasin D (MCE, HY-N6682, 10 μM). Preparation of Aβ-coated, pHrodo-labelled beads was performed as previously described (27). After addition of the beads, phagocytosis was monitored with the IncuCyte S3 live-cell analysis system by acquiring 3-4 brightfield and red fluorescence images every 45 minutes for 12h. For cell recognition and quantification of red-positive cells, the IncuCyte software with its adherent cell-by-cell classification tool was utilized.

### Molecular Phenotyping

Molecular phenotyping was performed as described previously (28–30). Expression levels of about 1,000 pre-selected “pathway reporter genes” are monitored using a targeted, parallelized, amplicon-based approach. Briefly, macrophages were treated for 4 hours with either anti-CD22 blocking antibody or the corresponding IgG control antibody at 5μg/ml. After incubation cells were lysed in 350 μl MagNA Pure LC RNA Buffer (Roche Diagnostics, 03604721001) and purified using the MagNA Pure 96 System (Roche Diagnostics). RNA was quantified using the Qubit RNA Assay Kit (Thermo Fisher Q32852) on the Fluorometer Glomax (Promega).

### Amplicon Library Construction

10 ng of total RNA from each biological replicate were reverse transcribed to cDNA using Super Script IV Vilo (Thermo Fisher, 11766500, user guide MAN0015862). Libraries were generated with the AmpliSeq Library Plus Kit (Illumina Catalog number 20019103) according to the Reference Guide. Pipetting Steps for target amplification, primer digestion and adapter ligation were done with the mosquito automatic pipettor (ttp labtech) in a miniaturisation fashion. For the purifications before and after final library amplification, SPRI magnetic bead purification was chosen (Clean NGS, LABGENE Scientific SA) semi automated on multidrop (Thermo Fisher). Amplicon size and DNA concentration were measured using an Agilent High Sensitivity DNA Kit (Agilent Technologies, Waldbronn, Germany) according to the manufacturer’s recommendation. Prior sequencing samples have to be normalized and pooled to 2nM final concentration on Biomek FXP workstation.

### Illumina Sequencing

Pooled libraries were sequenced on the Illumina NovaSeq 6000 Instrument, SBS (sequencing by synthesis) technology. 75 cycles end up with a minimum of 2 Mio SR per sample for analyzing.

### Read Mapping and Quantification

As every transcript is represented by a single, specific amplicon from the Molecular Phenotyping panel, the mapping results provide a direct quantification measure in “counts per amplicon” that is used for downstream analysis. Reads were mapped to the amplicon sequences of the panel using Bowtie2 (31).

### Detection of Differentially Expressed Genes, Gene Set Enrichment

Counts per amplicon were used for the downstream analyses, greatly simplifying the data processing. Differentially expressed genes (DEGs) were identified from the amplicon sequencing data using edgeR based on negative binomial distribution (28). Gene set enrichment was performed using CAMERA (32).

### Microglia Expression Modules

Microglia expression modules were derived from the publication of Friedman et al. (33) and complemented by two modules (DAM signatures TREM2 dependent and TREM2 independent) derived from the publication of Keren-Shaul et al. (34). Differences between two groups in these expression modules were visualized in a Radar plot using python.

### Statistics

GraphPad Prism was used for statistical analysis. Data sets were tested for normality (D’Agostino-Pearson Omnibus K^2^ normality test; significance level P=0.05) before employing the appropriate parametric or nonparametric statistical comparison test, which is depicted in the respective figure legend. If not stated otherwise, mean values ± SEM were reported. For outlier recognition, the Robust regression and Outlier removal (ROUT) method was utilized with an aggressiveness of Q=1. The Pearson correlation analysis was used to assess correlation.

## Results

### CD22 Expression Is Enhanced in Aged Microglia and Microglia With Internalized Aβ

**Figure 1:**
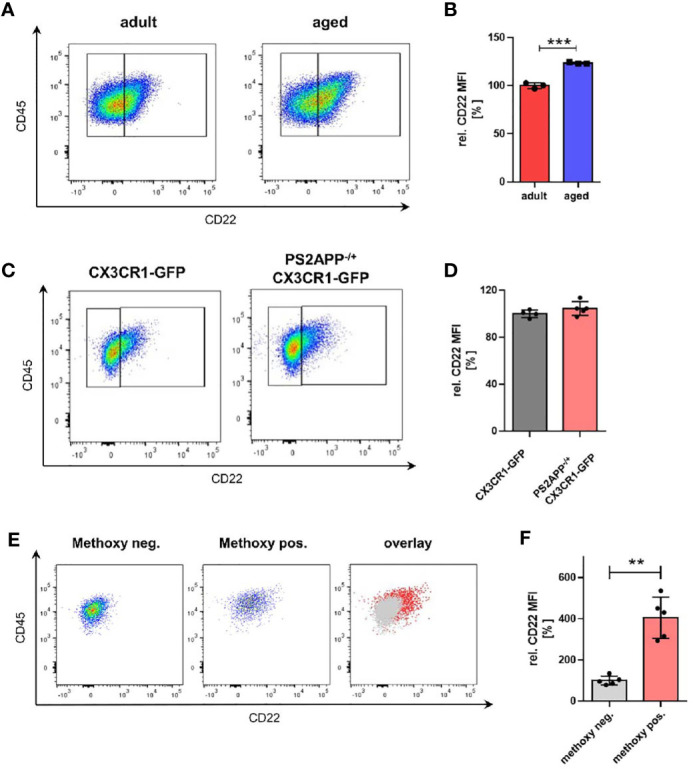
Microglia from aged mice as well as Aβ-containing microglia exhibit an increased CD22 expression. **(A)** Representative flow cytometry scatter plots and **(B)** respective quantification showing the relative CD22 MFI of isolated microglia from adult (6 months) and aged (14-16 months) mice. n=3 mice per age. **(C)** Representative scatter plots and **(D)** quantification of the CD22 expression in isolated microglia from 13 months old PS2APP^-/+^ CX3CR1-GFP and age-matched CX3CR1-GFP mice. n=4-5 mice. **(E)** Scatter plots of CD22 expression in methoxy-X04 negative microglia, methoxy-04 positive microglia and an overlay visualizing the differences in CD22 signal between both subsets. **(F)** Relative CD22 MFI of methoxy-X04 negative and methoxy-04 positive microglia from 13 months old PS2APP CX3CR1-GFP mice. n=5, mean ± SEM. For **(B, D)** and **(F)** mean ± SEM, unpaired nonparametric t-test (Mann-Whitney). **p<0.01, ***p<0.001.

During aging, microglia undergo significant transcriptional changes (11). However, the consequences for microglial physiology due to age-associated altered expression levels and implicated signaling pathways are not well understood yet. To address the role of microglial CD22 during aging, we started as a first step by comparing microglial CD22 expression between adult (6 months) and aged (14-16 months) mice. Microglia were isolated from CX3CR1-GFP reporter mice, sorted by flow cytometry and mean fluorescent intensity were measured to determine CD22 levels (**Figure S2A**). To exclude any potential impact of autofluorescence signal in the CD22 channel, a FMO (fluorescence minus one, including all antibodies except anti-CD22) control was included on pooled samples and used to set the gate for CD22 signal (**Figure S2B**). In 14-16 month aged mice, we found the level of CD22 in microglia to be significantly upregulated compared to the adult animals at 6 month of age (**Figures 1A, B**). With this observation we strengthen not only previous findings (15), but also narrow down the time window, in which an evident increase in CD22 expression can be observed.

Since also distinct transcriptional microglia states e.g. DAMs (disease-associated microglia) have been identified in amyloid mouse models of age-related neurodegenerative disorders (34), we wondered whether CD22 is differentially expressed in these subpopulations. To evaluate the relevance of CD22 in an AD-related context, we used the transgenic amyloid mouse model PS2APP (20). We compared CD22 expression of isolated microglia from aged-matched CX3CR1-GFP and PS2APP CX3CR1-GFP double-transgenic mice by flow cytometry, but did not observe a change in mean CD22 levels of all GFP-positive isolated microglia (**Figures 1C, D**). Since DAMs are often located in the vicinity of, or associated to Aβ-plaques, we injected PS2APP CX3CR1-GFP mice with methoxy-X04 prior to microglia isolation to label intracellular Aβ-material (35). When discriminating between microglia subpopulations in the PS2APP CX3CR1-GFP mice, Aβ-containing microglia (methoxy-positive cells) exhibited a significant 4-fold increase in CD22 levels in comparison to the methoxy-negative cells (**Figures 1E, F**), indicating an increased expression of CD22 in plaque-associated microglia.

Next, we were interested in how the increase in CD22 expression in aged and Aβ-containing microglia correlates with the physiological function, and more specifically surveillance capacity of these states.

### Two-Photon Imaging Based Slice Assay to Study Microglia Dynamics Upon CD22 Blockage

**Figure 2:**
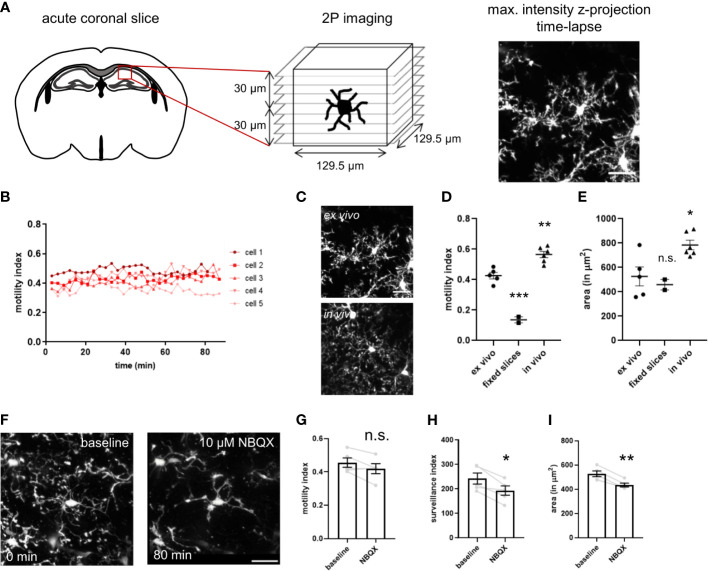
Imaging setup of two-photon microscopy of *ex vivo* acutely prepared slices. **(A)** Freshly prepared acute coronal sections of CX3CR1-GFP mice are placed under the microscope and the region of interest is selected. After adjusting the parameters for stack acquisition, the time-lapse stack recording is initiated. **(B)** The motility index, calculated for each timepoint, is stable for a 80 min imaging period. **(C)** Representative images of microglia process complexity in the *ex vivo* and *in vivo* preparation **(D)** Motility index and **(E)** cell area of microglia imaged in *ex vivo* slices, PFA-fixed slices and *in vivo* through a cranial window. **(F)** The AMPA receptor blocker NBQX induces progressive decrease in size and complexity of the ramified processes. NBQX treatment leads to a marginal reduction in motility **(G)**, and a more severe decrease of the surveillance index **(H)** and microglia area **(I)**. For **(D, E)** and **(G–I)** One-way ANOVA multiple comparisons to *ex vivo*, *p<0.05, **p<0.01, ***p<0.001, n.s.>0.5 Scale bar: 20 µm.

Microglia are continuously surveilling their parenchymal environment with their highly branched and motile processes (**Video S3**). They are extremely sensitive to small alterations in their surrounding environment and change their phenotype dramatically after isolation from the tissue (17, 18). Therefore, to assess microglial phenotypic modulation by CD22, it is essential to develop assays that are as close as possible to the natural microglial environment. In order to measure CD22 and other compound-mediated effects on microglia surveillance and motility, we performed live imaging by two-photon microscopy in *ex vivo* acute slices (**Figure 2A** and **Video S4**) of the CX3CR1-GFP transgenic mouse model (19). We have established a semi-automated image processing and analysis pipeline to quantify microglia dynamics (**Figure S1** and methods).

To determine if continuous imaging by the pulsed laser induces alterations in microglia dynamics, we have measured motility of single microglia over a period of 80 minutes (**Figure 2B**). We did not observe any change with stable motility values over time, indicating that the continuous imaging does not lead to tissue damage and associated microglia response. To evaluate the noise of our image processing and analysis pipeline, we have imaged PFA-fixed acute slices in the imaging chamber. The motility index is strongly reduced in fixed slices in comparison to *ex vivo* acute slices (**Figure 2D**). The residual minimal motility signal results from slice movement in the imaging chamber due to continuous flow of ACSF. Interestingly, microglia imaged *in vivo* through a cranial window in anesthetized animals revealed a higher motility index as compared to the *ex vivo* acute slices (**Figure 2D** and **Video S5**). The high sensitivity of microglia morphology and motility to spontaneous neuronal activity and neurotransmission (36, 37), that is reduced in the slice preparation, could explain these findings and correlates with the broader branched processes and increased covered area of microglia *in vivo* (**Figures 2C–E**).

For evaluation of our assay and assessment of its capability to detect microglial morphological changes, we used in a first step compounds to modulate microglial behavior. NBQX, an AMPA receptor antagonist, let to a slight drop in motility and more apparent in the surveillance index and occupied area (**Figures 2G–I**), and was concomitant with the observed de-ramification (**Figure 2F**) as seen in the time-lapse (**Video S6**). The sensitivity of the assay was further validated using the cytoskeleton blocker cytochalasin B and ATP (**Figure S3** and **Video S7**, **S8**). Taken together, we have established a microglia imaging and analysis platform that enables us to study their process dynamics in native tissue environment of the *ex vivo* slice preparation.

### Microglia of Aged Mice Show Reduced Surveillance Capacity

**Figure 3:**
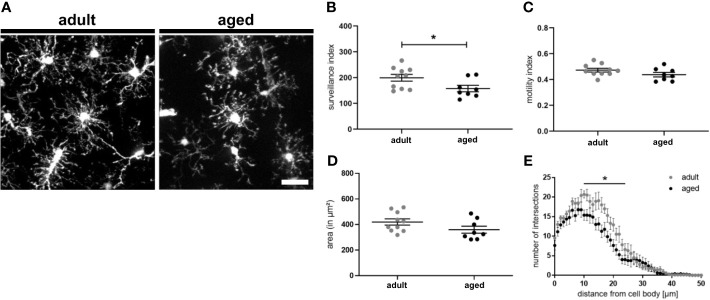
Surveillance deficits and hypo-ramification in aged microglia. **(A)** Representative two-photon images of microglia from acute slices of adult (7-8 months) and aged (13-16 months) mice. Semi-automated analysis of **(B)** surveillance index, **(C)** motility index and **(D)** area of adult and aged microglia. **(E)** Sholl analysis using max. z-projections from time point t_0_ of respective time-lapse recording showing the number of intersections in relation to the distance of the cell body. n=8-10 cells from n=7 mice each, mean ± SEM, unpaired nonparametric t-test (Mann-Whitney), *p<0.05. Scale bar: 20 µm.

Since we observed an upregulation of CD22 in microglia during aging and to address the question if CD22 is involved in age-related alterations of microglia physiology, we first compared dynamics of adult and aged microglia with the established two-photon based live imaging assay. We have prepared acute brain slices from 7-8 month old (adult) and 13-16 month old (aged) CX3CR1-GFP reporter mice. Microglia were live-imaged and the average surveillance, motility and area was calculated. Aged microglia exhibited a hypo-ramification as compared to microglia imaged from adult mice (**Figures 3A–E** and **Video S9**, **S10**). Strikingly, the hypo-ramification of aged microglia is accompanied by a significant reduction in their surveillance index as compared to adult microglia (**Figure 3B**). Microglia motility and cell area was reduced by 7% or 12%, respectively, but statistically there was no significant difference between the two groups (**Figures 3C, D**). Lastly, Sholl analysis reveals a significant reduction in ramification of aged compared to adult microglia (**Figure 3E**), thereby reflecting the age-associated morphological changes. These results show that aging of microglia is accompanied by a hypo-ramification and reduced surveillance of their parenchymal microenvironment.

### Antibody-Induced CD22 Blockage Restores Age-Related Decline in Microglial Surveillance

**Figure 4:**
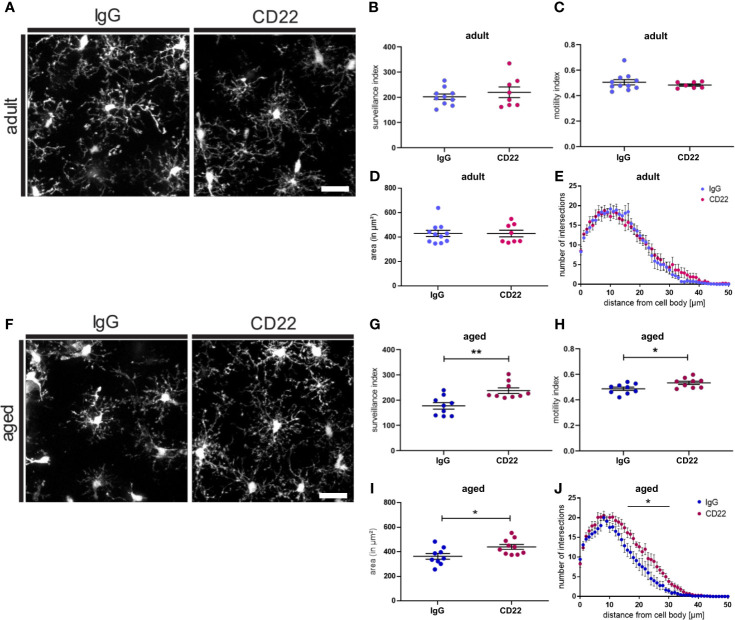
Blockage of CD22 restores age-associated deficits in surveillance and ramification. **(A)** Representative two-photon images of microglia from acute slices prepared from adult (7-8 months) mice, which were either treated with a CD22 or an IgG isotype control antibody. Respective analysis of the **(B)** surveillance index and **(C)** motility index as well as the **(D)** area and the **(E)** sholl analysis reflecting the microglial ramification. **(F)** Two-photon images of microglia from aged (13-16 months) mice demonstrating the effect of treatment with CD22-blocking antibody. Quantification of the **(G)** surveillance index **(H)** motility index, **(I)** area and **(J)** sholl analysis comparing the antibody treatments. For **(B–E)** and **(G–J)** n=7-11 cells from n=7 mice each, mean ± SEM, unpaired nonparametric t-test (Mann-Whitney), *p<0.05, **p<0.01. Scale bar: 20 µm.

CD22 deficiency leads to an enhanced motility as well as enhanced chemotaxis of B-cells (38). Therefore, we were asking if blockage of CD22 can also alter the morphology and dynamic activity of microglia. Since we observed a CD22 upregulation during aging of microglia, we were specifically interested if there is a differential effect on adult versus aged microglia. Thus, acute brain slices from adult (7-8 month old) and aged (13-16 month old) CX3CR1-GFP reporter animals were incubated with a CD22-blocking antibody or an isotype control antibody prior to imaging (**Figures 4A–F**). In adult microglia, antibody-mediated CD22 blockage did neither alter surveillance, motility and cell area, nor the ramification state as compared to IgG isotype control (**Figures 4B–E** and **Video S11**, **S12**). However, the evident de-ramification in aged microglia was reverted upon CD22 inhibition (**Figures 4A–J** and **Video S13**, **S14**) and an increase in overall cell area was observed (**Figure 4I**). Furthermore, the CD22-related morphological changes were accompanied by a significant increase of both surveillance and motility (**Figures 4G, H**). Ultimately, Sholl analysis revealed that microglial process ramification was restored by CD22 inhibition (**Figure 4J**). These results clearly demonstrate that in accordance with the upregulation of CD22 during aging, age-associated de-ramification and declining surveillance capacity can be restored *via* antibody-mediated inhibition of CD22 specifically in aged microglia and suggest CD22 as a negative regulator of microglia surveillance.

In addition to aging, we also observed a specific upregulation of CD22 in Aβ-containing microglia in the amyloid mouse model PS2APP CX3CR1-GFP. Extracellular accumulation of Aβ, a pathological hallmark of Alzheimer’s disease, is associated with neuroinflammation and aberrantly activated microglia, referred to as disease-associated microglia (DAM) (34), that are specifically found around plaques. These DAMs exhibit a modified transcriptional signature, such as the upregulation of AD risk genes and the downregulation of homeostatic genes. Furthermore, plaque-associated microglia exhibit a reduced process motility (39). We therefore tested if CD22 blockage can also alter surveillance and motility of plaque-associated microglia. To analyze the effects of CD22 inhibition on plaque-associated microglia, two-photon live imaging was performed on brain slices prepared from methoxy-X04 injected PS2APP CX3CR1-GFP animals to label the plaques (**Figure S4A**). Since this amyloid mouse model develops plaques first in the frontal cortex, we selected this region for further evaluation. In contrast to homeostatic microglia, plaque-associated microglia exhibit a reduced surveillance and motility index of around 73 and 0.4, respectively. Upon blockage with the CD22 antibody, the surveillance index increased to 85, but statistically there was no significant difference to the IgG isotype control (**Figure S4B**). Motility of plaque-associated microglia was not affected by CD22 inhibition (**Figure S4C**). Although CD22 expression is strongly increased in methoxy-positive microglia, antibody-mediated blockage of CD22 did not significantly alter the surveillance of plaque-associated microglia.

Summarizing, with two-photon imaging of microglial dynamics in acutely prepared slices we show that the inhibition of CD22 signaling can enhance microglial motility as well as restore age-related decline in microglia surveillance and ramification in the non-diseased brain. In a disease context, plaque-associated microglia did not display significant changes upon antibody-mediated blockage of CD22, although a trend towards an increased surveillance capacity is visible.

### CD22 Blockage Enhances Phagocytosis of Aβ-Coated Beads in Human iPSC-Derived Macrophages

**Figure 5:**
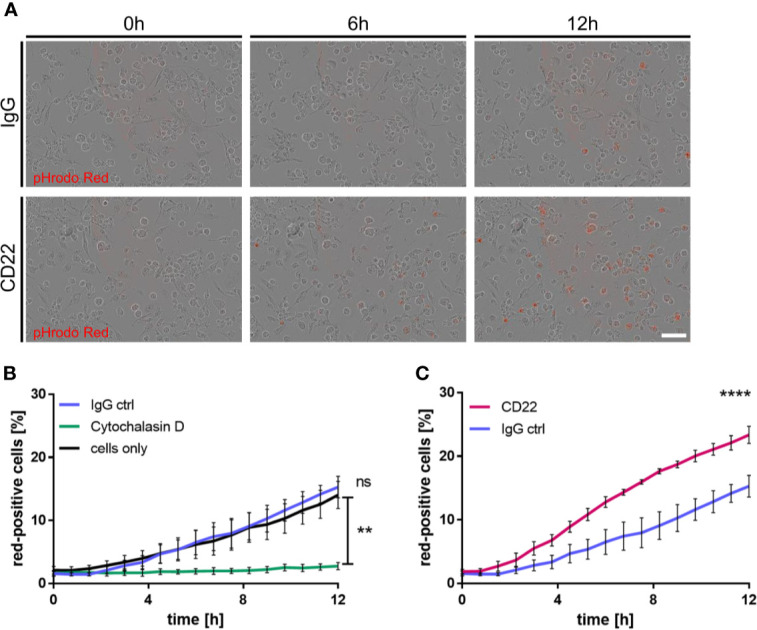
CD22 blockage increases phagocytic capacity of human iPSC-derived macrophages. **(A)** Representative images of iPSC macrophages treated either with CD22 blocking antibody or IgG isotype control. Red signal indicates pH-rodo labeled Aβ-coated beads, which are located in the lysosomes. **(B)** Increase of phagocytosis-positive cells over time in untreated cells and cells treated with 5 μg/ml IgG isotype control, while no significant change is visible in cells treated with 10 μM Cytochalasin D. Cells were monitored using IncuCyte S3 and analyzed using IncuCyte cell by cell analysis. **(C)** Raise in phagocytosis positive cells over time in cells treated with 5 μg/ml CD22 blocking antibody or 5 μg/ml IgG isotype control. Cells were monitored using IncuCyte S3 and analyzed using IncuCyte cell by cell analysis. n=3, mean ± SEM, unpaired nonparametric t-test (Mann-Whitney), **p<0.01, ****p<0.0001. ns>0.05.

CD22 has recently been identified as a modulator of microglial phagocytosis in a CRISPR screen based on Cas9 expressing murine BV2 cells (15). To connect observations from this study and our mouse *ex vivo* slices to human biology, we derived primitive macrophages from iPSC cells and assessed the effect of CD22 blockage on phagocytic activity. For the generation of the iPSC-derived macrophages we used a previously published protocol (25, 26) that resembles the correct ontogeny of yolk-sac-derived myb-independent primitive macrophages. Since macrophages are highly phagocytic cells with similar molecular mechanisms involved in phagocytosis as in microglia and we already established a variety of functional readouts using these cells previously we decided to use these cells as surrogate for microglial phagocytosis. To assess the phagocytic activity of the macrophages, we monitored the uptake of pHrodo-labelled Aβ-coated beads (27), which were added to the respective treatments. Upon treatment with the IgG isotype control antibody the amount of red-positive cells, which reflect the amount of phagocytic cells, does not differ over time compared to the control condition, whereas the phagocytic activity is completely abolished by cytochalasin D, a known actin polymerization inhibitor (**Figure 5B**). However, antibody-mediated CD22 blockage leads to a significant increase in red-positive cells over time compared to the isotype control (**Figures 5A–C**), indicating an increased phagocytic activity. Thus we conclude that CD22 negatively regulates phagocytosis in human macrophages *in vitro*, similar to previous findings reported in murine microglia-like cells (15).

### CD22 Blockage Effects on Gene Expression in Human iPSC-Derived Macrophages

**Figure 6:**
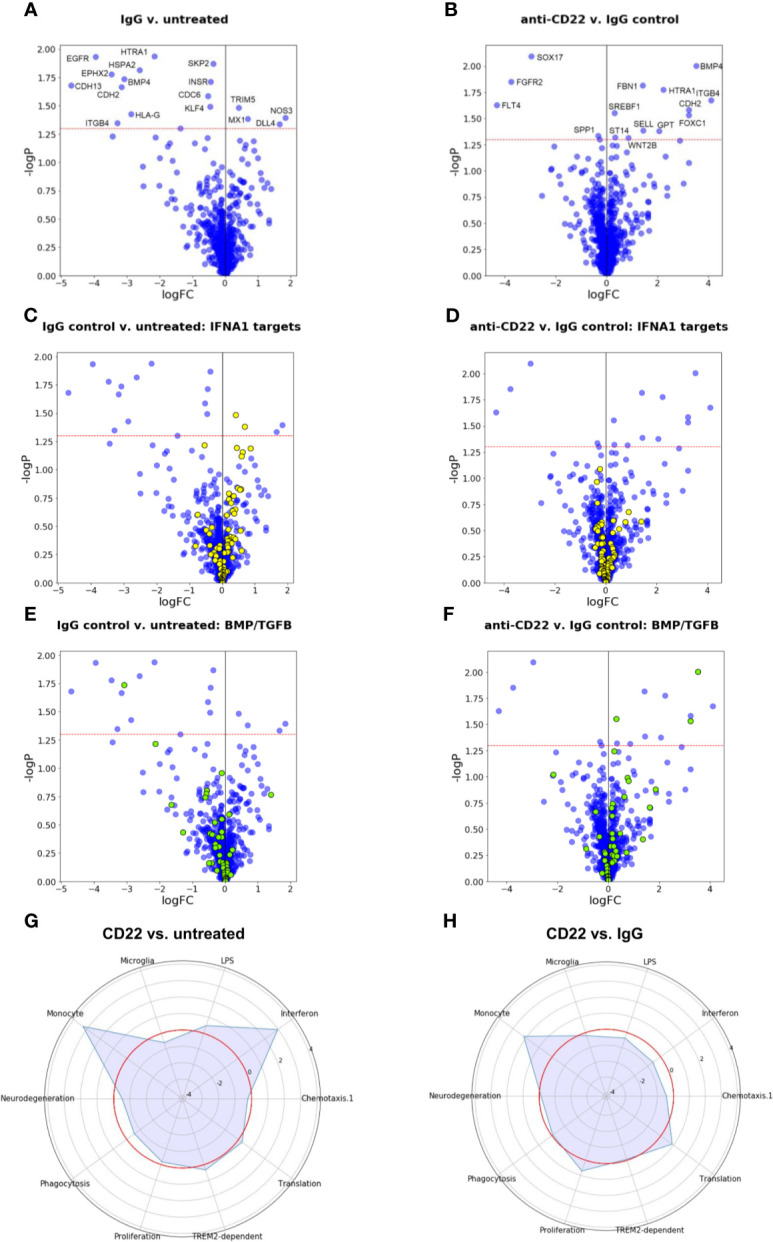
Gene expression and gene set enrichment analyses. **(A, B)** Volcano plots of treatment effects on gene expression levels in iPSC-derived macrophages. Each point represents one of the 1,062 measured pathway reporter genes. The x axis shows log2 fold changes, the y axis has negative log10 of p values. The red horizontal line indicates a p value cutoff of 5 percent. All genes above the cutoff are indicated by their official gene symbols. **(A)** Effects of generic IgG antibody on untreated cells. **(B)** Effects of anti-CD22 antibody treatment compared to IgG treatment. **(C, D)** Volcano plots as in **(A, B)**, with genes annotated as targets of interferon alpha (IFNA) highlighted in yellow. **(E, F)** Volcano plots as in **(A, B)**, with genes belonging to BMP/TGFB signaling highlighted in yellow. **(G)** Radar plot to visualize changes in gene expression in gene modules relevant for microglia induced by CD22 blockage. Plotted values are enrichment scores calculated using CAMERA (32). The red circle indicates the reference level as detected in untreated macrophages. Peaks outside the red circle indicate up-regulation of a module in cells treated with CD22 blocking antibody and modules closer to the center indicate down-regulated modules compared to untreated macrophages. **(H)** Radar plot to visualize changes in gene expression in microglial modules induced by CD22 blockage in comparison to Isotype treated cells. The red circle indicates the reference level as detected in Isotype treated macrophages.

Since the observation of increased phagocytosis pointed to a specific effect of CD22 blockage on macrophages we used molecular phenotyping (28–30) to further characterize downstream pathways that are affected by CD22 blockage. **Figures 6A, B** shows volcano plots for the comparisons of control IgG-treated cells to untreated cells, and of anti-CD22-treated cells to IgG-treated cells. The horizontal red dashed line corresponds to a p value cutoff of p=0.05. Even for a panel of around 1,000 genes, the number and size of effects at the transcriptional level are small, and are comparable for the two tested treatments. Obviously, signaling events at the protein level are not captured in a differential gene expression analysis. We note that effects on phagocytosis and motility may well be regulated at the protein level and not be reflected in the gene expression data. **Figures 6A, B** shows that several of the genes that are down-regulated in response to the generic IgG antibody (left: ITGB4, BMP4, HTRA1, CDH2) are up-regulated by anti-CD22 treatment compared to IgG.

Given the small number of clearly significant effects, we apply a gene set enrichment analysis approach to identify effects that are significant at the gene set level even though non-significant at the gene level. We assessed a total of about 3,000 gene sets obtained from the public domain as described in (28–30). **Figures 6C–F** illustrates two very clear findings.

First, the interferon response network is characterized here by annotated targets of interferon alpha (IFNA1). As the diagram in **Figure 6C** shows, for IgG controls, two IFNA1 target genes are up-regulated at the p=0.05 significance level (TRIM5, MX1, compare **Figure 6A**), but the majority of other IFNA1 target genes also show an upward trend, rendering this gene set as a whole significantly up-regulated. In **Figure 6D**, the same gene set is shown for the anti-CD22 treatment compared to IgG treatment. The set is overall balanced, and none of the member genes is significantly affected anymore, and there is no overall effect on this set of genes, suggesting that the anti-CD22 effect in this context alleviated the interferon response induced by the control antibody.

For another gene set of interest, genes annotated as involved in BMP/TGFB signaling, the situation is even more dramatic. **Figure 6E** suggests that, while only one of its member genes (BMP4) is statistically significantly down-regulated by treatment with IgG, the majority of member genes measured in our panel show a downward trend. As **Figure 6F** shows, this trend is reversed by treatment with anti-CD22. The majority of BMP/TGFB signaling genes now show upward trends, and three of them are individually significant at the p=0.05 level (SREBF1, FOXC1, BMP4). TGF-β signaling was associated with homeostasis of microglia (40).

For comparison with our previous work, we also used the previously described microglial gene modules (27) and compared the effect of CD22 blockage on these modules to untreated cells and isotype control antibody treated cells (**Figures 6G, H**). We note that CD22 itself is part of the “neurodegeneration” module analyzed by Friedman et al. (33). When compared to untreated cells, an up-regulation of the monocyte module and the interferon module could be observed (**Figure 6G**). As explained in more detail above (**Figures 6C, D**), the effect on the interferon module was not observed when the CD22 blockage was compared to isotype control. However, the monocyte module was still affected.

Overall, while we do not observe any clear gene expression effects related to phagocytosis or cell motility, blockage of CD22 signaling is shown to affect gene networks involved in modulating overall microglia activation and behavior.

## Discussion

Here we describe a novel function of microglial expressed CD22 in regulating process surveillance and ramification. We have assessed microglial motility, surveillance and ramification *ex vivo* in acutely prepared brain slices using two-photon microscopy. Utilizing this technology, we were able to demonstrate age-related decrease in microglial surveillance and altered morphology comparing microglia from adult and aged CX3CR1-GFP reporter mice, which are consistent with the de-ramification of aged microglia observed in human studies (41). Importantly, the blockage of CD22 in acute brain slices of aged mice restored age-related microglial hypo-ramification and the reduced surveillance capacity. Moreover, CD22 blockage results in increased phagocytosis of Aβ-coated beads in iPSC-derived macrophages and reprograms transcriptional state inducing an upregulation of BMP/TGFβ gene networks and a dampening of the interferon response.

### Two-Photon Microglia Imaging

To evaluate dynamic and morphological changes in microglia upon blockage of CD22, we describe a two-photon imaging and analysis platform. While already various approaches were reported to study microglia dynamics *in vivo*, most of them are dependent on manual selections, which are more prone to bias and which we aimed to avoid with our semi-automated pipeline. An additional advantage of our analysis platform is its compatibility with both *in vivo* and *ex vivo* imaging, making it more versatile. A recently published review summarized different approaches on studying microglia dynamics (42) and in contrast to our data, when comparing the microglial process speed of different conditions, published data suggests an enhanced process speed in *ex vivo* acute slice recordings compared to *in vivo* imaging. However, the authors also illustrate that caution has to be taken by comparing values from different studies since the experimental settings often differ. We observe increased motility by *in vivo* imaging in anesthetized mice compared to *ex vivo* acute brain slices with the same acquisition and analysis pipeline, which is in line with a higher complexity of the microglial processes *in vivo*. Since our platform can be utilized for an *in vivo* and *ex vivo* setup, the obtained data is more comparable. Overall, this platform does not only help to further understand microglia dynamics and physiology, but also allows to dissect molecular pathways which control phenotypic alterations and subsequently to develop new therapeutic strategies to manipulate microglia behavior.

### Aging and CD22

Numerous studies are linking aging with microglia deficits, making further research in understanding those impairments and their relevance for neurodegeneration indispensable. Our study for instance shows that CD22 blockage also restores the surveillance capacity and ramification of aged microglia in addition to the previously published effects on age-associated suppression of microglial phagocytosis (15). Since we and others have shown that murine CD22 levels are upregulated during aging (43), this potentially uncovers CD22 as a broad age-related modifier of microglial behavior. While we were able to show significant age-associated reductions in surveillance and ramification, only a trend towards a reduced area and motility was observed. Although previous studies robustly demonstrated age-related changes in microglial motility (42), it is important to emphasize that the age difference in our study is intentionally small (adult versus aged) and therefore we expect that the effects would be even more prominent with bigger age differences. Nevertheless, we provide valuable data on how to potentially modulate microglial malfunction observed during aging.

### CD22 and Neurodegeneration

Despite RNASeq data indicating an increased CD22 expression in AD brains (33), little is known about the presumed role of CD22 in neurodegeneration. However, CD33, a member of the SIGLEC-family and an AD risk-gene (44), was shown to impair microglial Aβ-uptake and clearance (45, 46). Moreover, a SNP in INPP5D, the gene coding for the CD22 downstream signaling partner SHIP-1, was associated with late-onset Alzheimer’s disease (LOAD) (47). By using the amyloid mouse model PS2APP, we evaluated the effects of CD22 blockage in an amyloid context. While CD22 overall expression comparing CX3CR1-GFP and PS2APP CX3CR1-GFP animals did not significantly differ, methoxy-positive microglia show considerably increased protein levels of CD22. This is in line with previously published scRNAseq data that show upregulation of CD22 mRNA in DAM state microglia (34). As plaque-associated microglia display an altered transcriptomic signature (34, 48), we could speculate that the uptake of Aβ or their transformation into a DAM state might cause an enhanced CD22 expression. While we observed clear morphological changes in aged microglia after blockage of CD22, data on process dynamics of plaque-associated microglia of PS2APP CX3CR1-GFP mice was more heterogeneous. The transcriptional signature of microglia is highly diverse between homeostatic versus plaque-associated microglia and throughout the different brain regions (11). This heterogeneity and differential expression of CD22 signaling partners could generally influence the susceptibility of microglia for targeted inhibitions like CD22 blockage. Since the plaque-associated microglia of PS2APP CX3CR1-GFP were imaged exclusively in the frontal cortex, evaluating the potential region-specificity of effects on microglial CD22 blockage might therefore be of interest for future experiments. Nevertheless, the option remains, that CD22 is mainly an age-associated modifier of microglial surveillance rather than disease-associated.

### CD22 Downstream Signaling and Molecular Phenotypes

Transcriptomics studies emphasize differences between human and mouse microglia during aging and Alzheimer’s disease (9, 49), underlining the importance of translational studies with human tissue or cells. By demonstrating a modulatory effect of CD22 blockage on Aβ phagocytosis in human iPSC-derived macrophages, we show the relevance of CD22 targeting for human biology. Further dissection of the microglial CD22 pathway will be necessary to fully understand the observed effects upon its inhibition, for instance elucidating the mechanisms underlying the increased CD22 expression during aging or the direct downstream effects on intracellular signaling pathways. The CD22 intracellular domain consists of ITIMs (immunoreceptor tyrosine-based inhibition motif), which, by recruiting the protein tyrosine phosphatase SHP-1, can counteract signals arising from ITAM (immunoreceptor tyrosine-based activation motif) receptors (50). DAP12, the TREM2 adaptor protein, presents an example of ITAM containing receptors in microglia and latest research indicates that ITAM signaling plays an important role in the phagocytosis process (51). We have observed an increased phagocytosis upon CD22 blockage, which might be mediated by disinhibition and the release of ITAM signaling. Especially interesting would be the association with TREM2 signaling, which has been identified as a key receptor promoting microglial phagocytosis (52). Indeed, CD33, another ITIM siglec receptor, opposes TREM2 signaling and inhibits phagocytosis (46).

In addition to increased phagocytosis, we have also observed that inhibition of CD22 leads to enhanced surveillance and motility of microglia. A higher overall cell motility could also explain the finding of increased phagocytosis. CD22 signaling pathways have been characterized in other cell types including B-cells where it regulates B-cell receptor signaling *via* recruiting protein tyrosine phosphatase SHP-1 and SHP-2 (50). They in turn modulate the function of various signaling pathways and thereby control several cellular functions like chemotaxis or inflammation (53). Both SHP-1 and SHP-2 are also highly expressed in microglia (53), which suggest CD22 signaling *via* these protein tyrosine phosphatases during microglial phagocytosis as well. For instance, inhibition of SHP-1 was shown to rescue α-synuclein-associated inhibition of phagocytosis in rat primary microglia (54).

Here we already took one further step in dissecting the effects of CD22 blockage, by analyzing its transcriptional effects on human iPSC-derived macrophages. As expected for non Fc-silent antibodies, the CD22 blocking antibody as well as its isotype control showed some overlapping effects on signaling networks (55, 56). Overall, differential effects on a panel of “pathway reporter genes” were mild, but gene set enrichment analysis revealed interesting network-level effects. Strikingly, in comparison to previously published RNAseq on CD22 inhibition, we also observed an enrichment in TGF-β signaling (15). CD22 could influence the balance between the two major microglial survival pathways, the MCSF and TGF-β signaling (57). The latter, upregulated by CD22 blockage in our study, has previously been reported to influence microglial morphology (58). Therefore, the transcriptional effects observed in the human macrophages correlate with our observation of the morphology changes in the *ex vivo* slices. Furthermore, TGF-β signaling in microglia is central in the prevention of excessive activation of mature microglia, whereas homeostatic gene expression signature is not affected by TGF-β (59). Microglial activation is generally considered to be associated with aging. Thereby, CD22-blockage induced TGF-β signaling could present a mechanistic link of the observed age-dependent effects of CD22 inhibition on microglial surveillance. In addition we clearly saw an effect of CD22 blockage dampening the IgG-induced interferon response. *In vivo*, the microglia interferon response drives microglia away from the homeostatic stage towards a pro-inflammatory stage (33). The interferon module was found to be induced in disease and CD22 could play a role in inducing this module.

Summarizing, we have identified CD22 as a novel age-related modifier of microglial surveillance. The significance of CD22 signaling in human biology was confirmed in iPSC-derived macrophages, where CD22 blockage increases phagocytosis and modulates key gene networks implicated in microglial function.

## Data Availability Statement

The datasets presented in this study can be found in online repositories. The names of the repository/repositories and accession number(s) can be found below: https://www.ncbi.nlm.nih.gov/geo/, GSE169252.

## Ethics Statement

The animal study was reviewed and approved by the Cantonal Ethical Committee for Animal Research and the Swiss Cantonal Veterinary Office.

## Author Contributions

EH and SG conceived, designed, and supervised the study. EH and CC-B established the two-photon imaging-based brain slice assay. VA conducted the *ex vivo* imaging experiments. AP and VA performed the flow cytometry and AP analyzed the data. VA and CS did the cellular experiments. ME, RS, and EK conducted the molecular phenotyping analysis and evaluated the data. VA drafted the article and co-wrote the paper with SG and EH. CC-B, AP, ME, RS, CS, and EK revised the article critically. All authors contributed to the article and approved the submitted version.

## Funding

VA was supported by the Roche Internships for Scientific Exchange (RiSE) program.

## Conflict of Interest

During the course of this study, all authors are or were full time employees or trainees at Roche and they may additionally hold Roche stock/stock options.

The reviewer DE declared a shared affiliation with one of the authors VA to the handling editor at the time of the review.

## Supplementary Material

Supplementary Figure 1 Image processing and quantification of two-photon recordings. **(A)** A maximum intensity projection of the acquired stacks for each timepoint is used to generate the time-lapse. After x,y registration and manual cropping of the cell, an automated series of filters for image correction is applied for thresholding and binarization of the images. **(B)** To assess the process retractions, image tn is subtracted with tn+1. The results represent the amount of processes in tn which retracted in the following image tn+1. For the process extensions, image tn is subtracted from tn+1, describing the amount of processes which extended from tn to tn+1. While the motility index indicates the microglia process dynamics, the surveillance index serves as a readout for microglial surveillance. Correlation plots of the **(C)** relative motility, **(D)** relative surveillance and **(E)** relative area against the time after slice preparation to evaluate the stability of the measured readouts over time in untreated adult and aged microglia, which were used for experiment in Figure 3.

Supplementary Figure 2 Supplementary flow cytometry data. **(A)** Gating strategy to assess microglial CD22 expression. Cells were gated based on their location on the FCS-A vs SSC-A plot and doublets were excluded consequently (FSC-A vs FCS-H). Live single cells were assessed for CD45 positivity and myeloid cells were gated (CD11b^+^CD45^+^ cells). Finally, CD22 expression in CX3CR1 positive myeloid cells (GFP+ cells) was analyzed. **(B)** Scatter plots to visualize acquired CD22 signal per sample. FMO: Fluorescence Minus One. As control, one sample was stained with all antibodies except the CD22-PE. This sample was used to set the CD22 negative and positive gates, which was then applied on all samples.

Supplementary Figure 3 Application of other known microglia modulators induce distinct dynamic responses. **(A)** Wash-in of the actin polymerization inhibitor cytochalasin B diminishes the process speed **(B)** and the surveilled area **(C)**, while the total area remains stable **(D)**. **(E)** ATP evokes an increase in size and complexity of the processes and the development of bulbous tips. Upon application of ATP, a reversible increase of motility **(E)** and surveillance **(F)** followed by a recovery to steady state, whereas a constant increase of microglia area was measured **(G)**. n=5-6 cells per treatment. Mean ± SEM, (B)-(D) paired t-test, (F)-(H) one-way ANOVA multiple comparisons to baseline, * p<0.05, ** p<0.01, n.s.>0.05. Scale bar: 20 µm.

Supplementary Figure 4 Plaque-associated microglia trend towards an increased surveillance capability upon CD22 blockage. **(A)** Representative two-photon images of plaque-associated microglia in 8-12 months old PS2APP x CX3CR1-GFP mice, which were either treated with a CD22 or an IgG isotype control antibody. Respective surveillance **(B)** and motility **(C)** analysis. n=8 cells from n=8 mice, mean ± SEM, unpaired nonparametric t-test (Mann-Whitney), Scale bar: 20 µm.

Supplementary Video 1 Time-lapse of activated microglia at the slice surface. Scale bar: 20 µm.

Supplementary Video 2 Two-photon Z-scan through the acute slice, starting at the slice surface until a depth of -111 µm. Scale bar: 20 µm.

Supplementary Video 3 Montage of time-lapse (left) and cumulative surveilled area over time (right) of a homeostatic microglia. Scale bar: 20 µm.

Supplementary Video 4 Two-photon recording of homeostatic microglia at a depth of -80 µm. Scale bar: 20 µm.

Supplementary Video 5 Two-photon time-lapse of homeostatic microglia in vivo. Scale bar: 20 µm.

Supplementary Video 6 10µM NBQX provokes a severe microglial de-ramification when applied on the acute slice. Scale bar: 20 µm.

Supplementary Video 7 Time-lapse of microglia experiencing abolishment of process dynamics upon wash-in of 1µM cytochalasin B. Scale bar: 20 µm.

Supplementary Video 8 During wash-in of 1mM ATP, microglia react immediately by hyper-ramification and temporal development of bulbous tips. Scale bar: 20 µm.

Supplementary Video 9 Two-photon recording of microglia from an adult mouse under steady-state conditions. Scale bar: 20 µm.

Supplementary Video 10 Time-lapse of an aged microglia demonstrating age-associated hypo-ramification. Scale bar: 20 µm.

Supplementary Video 11 No evident differences can be observed in adult microglia after treatment with IgG antibody. Scale bar: 20 µm.

Supplementary Video 12 Upon pre-treatment with the CD22 antibody, no changes in microglia dynamics are visible in microglia from adult mice. Scale bar: 20 µm.

Supplementary Video 13 After IgG control antibody incubation, reduced ramification of aged microglia remains unaffected. Scale bar: 20 µm.

Supplementary Video 14 Antibody-mediated CD22-blockage reverses the age-related hypo-ramification of aged microglia. Scale bar: 20 µm.

## References

[1] Thion MS Ginhoux F Garel S. Microglia and Early Brain Development: An Intimate Journey. Science (2018) 362:185–9. 10.1016/j.stem.2010.08.014 30309946

[2] Sierra A Encinas JM Deudero JJP Chancey JH Enikolopov G Overstreet-Wadiche LS. Microglia Shape Adult Hippocampal Neurogenesis through Apoptosis-Coupled Phagocytosis. Cell Stem Cell (2010) 7:483–95. 10.1016/j.stem.2010.08.014 PMC4008496 20887954

[3] Bilimoria PM Stevens B. Microglia Function During Brain Development: New Insights From Animal Models. Brain Res (2015) 1617:7–17. 10.1016/j.brainres.2014.11.032 25463024

[4] Tremblay M Zettel ML Ison JR Allen PD Majewska AK. Effects of Aging and Sensory Loss on Glial Cells in Mouse Visual and Auditory Cortices. Glia (2012) 60:541–58. 10.1002/glia.22287 PMC3276747 22223464

[5] Wolf SA Boddeke HWGM Kettenmann H. Microglia in Physiology and Disease. Annu Rev Physiol (2016) 79:619–43. 10.1146/annurev-physiol-022516-034406 27959620

[6] Colonna M Butovsky O. Microglia Function in the Central Nervous System During Health and Neurodegeneration. Annu Rev Immunol (2016) 35:1–28. 10.1146/annurev-immunol-051116-052358 28226226 PMC8167938

[7] Chan A Magnus T Gold R. Phagocytosis of Apoptotic Inflammatory Cells by Microglia and Modulation by Different Cytokines: Mechanism for Removal of Apoptotic Cells in the Inflamed Nervous System. Glia (2001) 33:87–95. 10.1002/1098-1136(20010101)33:1<87::aid-glia1008>3.0.co;2-s 11169794

[8] Nimmerjahn A Kirchhoff F Helmchen F. Resting Microglial Cells Are Highly Dynamic Surveillants of Brain Parenchyma In Vivo. Science (2005) 308:1314–8. 10.1126/science.1110647 15831717

[9] Galatro TF Holtman IR Lerario AM Vainchtein ID Brouwer N Sola PR. Transcriptomic Analysis of Purified Human Cortical Microglia Reveals Age-Associated Changes. Nat Neurosci (2017) 20:1162–71. 10.1038/nn.4597 28671693

[10] Hefendehl JK Neher JJ Sühs RB Kohsaka S Skodras A Jucker M. Homeostatic and Injury-Induced Microglia Behavior in the Aging Brain. Aging Cell (2014) 13:60–9. 10.1111/acel.12149 PMC4326865 23953759

[11] Grabert K Michoel T Karavolos MH Clohisey S Baillie JK Stevens MP. Microglial Brain Region–Dependent Diversity and Selective Regional Sensitivities to Aging. Nat Neurosci (2016) 19:504–16. 10.1038/nn.4222 PMC4768346 26780511

[12] Sierra A Gottfried-Blackmore AC McEwen BS Bulloch K. Microglia Derived From Aging Mice Exhibit an Altered Inflammatory Profile. Glia (2007) 55:412–24. 10.1002/glia.20468 17203473

[13] Safaiyan S Kannaiyan N Snaidero N Brioschi S Biber K Yona S. Age-Related Myelin Degradation Burdens the Clearance Function of Microglia During Aging. Nat Neurosci (2016) 19:995–8. 10.1038/nn.4325 PMC7116794 27294511

[14] Hou Y Dan X Babbar M Wei Y Hasselbalch SG Croteau DL. Ageing as a Risk Factor for Neurodegenerative Disease. Nat Rev Neurol (2019) 15:565–81. 10.1038/s41582-019-0244-7 31501588

[15] Pluvinage JV Haney MS Smith BAH Sun J Iram T Bonanno L. CD22 Blockade Restores Homeostatic Microglial Phagocytosis in Ageing Brains. Nature (2019) 568:187–92. 10.1038/s41586-019-1088-4 PMC6574119 30944478

[16] Müller J Nitschke L. The Role of CD22 and Siglec-G in B-cell Tolerance and Autoimmune Disease. Nat Rev Rheumatol (2014) 10:422–8. 10.1038/nrrheum.2014.54 24763061

[17] Bohlen CJ Bennett FC Tucker AF Collins HY Mulinyawe SB Barres BA. Diverse Requirements for Microglial Survival, Specification, and Function Revealed by Defined-Medium Cultures. Neuron (2017) 94:759–73.e8. 10.1016/j.neuron.2017.04.043 28521131 PMC5523817

[18] Sierra A Paolicelli RC Kettenmann H. Cien Años De Microglía: Milestones in a Century of Microglial Research. Trends Neurosci (2019) 42:778–92. 10.1016/j.tins.2019.09.004 31635851

[19] Jung S Aliberti J Graemmel P Sunshine MJ Kreutzberg GW Sher A. Analysis of Fractalkine Receptor CX3CR1 Function by Targeted Deletion and Green Fluorescent Protein Reporter Gene Insertion. Mol Cell Biol (2000) 20:4106–14. 10.1128/mcb.20.11.4106-4114.2000 PMC85780 10805752

[20] Ozmen L Albientz A Czech C Jacobsen H. Expression of Transgenic APP Mrna Is the Key Determinant for Beta-Amyloid Deposition in PS2APP Transgenic Mice. Neurodegener Dis (2008) 6:29–36. 10.1159/000170884 19066434

[21] Nissen JC. Microglial Function Across the Spectrum of Age and Gender. Int J Mol Sci (2017) 18:561. 10.3390/ijms18030561 PMC5372577 28273860

[22] Villa A Torre SD Maggi A. Sexual Differentiation of Microglia. Front Neuroendocrin (2018) 52:156–64. 10.1016/j.yfrne.2018.11.003 30481522

[23] Thevenaz P Ruttimann UE Unser M. A Pyramid Approach to Subpixel Registration Based on Intensity. IEEE T Image Process (1998) 7:27–41. 10.1109/83.650848 18267377

[24] Klunk WE Bacskai BJ Mathis CA Kajdasz ST McLellan ME Frosch MP. Imaging Aβ Plaques in Living Transgenic Mice With Multiphoton Microscopy and Methoxy-X04, a Systemically Administered Congo Red Derivative. J Neuropathol Exp Neurol (2002) 61:797–805. 10.1093/jnen/61.9.797 12230326

[25] Gutbier S Wanke F Dahm N Rümmelin A Zimmermann S Christensen K. Large-Scale Production of Human iPSC-Derived Macrophages for Drug Screening. Int J Mol Sci (2020) 21:4808. 10.3390/ijms21134808 PMC7370446 32645954

[26] Wilgenburg BV Browne C Vowles J Cowley SA. Efficient, Long Term Production of Monocyte-Derived Macrophages From Human Pluripotent Stem Cells Under Partly-Defined and Fully-Defined Conditions. PloS One (2013) 8:e71098. 10.1371/journal.pone.0071098 23951090 PMC3741356

[27] Reich M Paris I Ebeling M Dahm N Schweitzer C Reinhardt D. Alzheimer’s Risk Gene TREM2 Determines Functional Properties of New Type of Human iPSC-Derived Microglia. Front Immunol (2021) 11:617860. 10.3389/fimmu.2020.617860 33613545 PMC7887311

[28] Zhang JD Schindler T Küng E Ebeling M Certa U. Highly Sensitive Amplicon-Based Transcript Quantification by Semiconductor Sequencing. BMC Genomics (2014) 15:565. 10.1186/1471-2164-15-565 24997760 PMC4101174

[29] Drawnel FM Zhang JD Küng E Aoyama N Benmansour F Rosario AAD. Molecular Phenotyping Combines Molecular Information, Biological Relevance, and Patient Data to Improve Productivity of Early Drug Discovery. Cell Chem Biol (2017) 24:624–34.e3. 10.1016/j.chembiol.2017.03.016 28434878

[30] Zhang JD Küng E Boess F Certa U Ebeling M. Pathway Reporter Genes Define Molecular Phenotypes of Human Cells. BMC Genomics (2015) 16:342. 10.1186/s12864-015-1532-2 25903797 PMC4415216

[31] Langmead B Salzberg SL. Fast Gapped-Read Alignment With Bowtie 2. Nat Methods (2012) 9:357–9. 10.1038/nmeth.1923 PMC3322381 22388286

[32] Wu D Smyth GK. Camera: A Competitive Gene Set Test Accounting for Inter-Gene Correlation. Nucleic Acids Res (2012) 40:e133–3. 10.1093/nar/gks461 PMC3458527 22638577

[33] Friedman BA Srinivasan K Ayalon G Meilandt WJ Lin H Huntley MA. Diverse Brain Myeloid Expression Profiles Reveal Distinct Microglial Activation States and Aspects of Alzheimer’s Disease Not Evident in Mouse Models. Cell Rep (2018) 22:832–47. 10.1016/j.celrep.2017.12.066 29346778

[34] Keren-Shaul H Spinrad A Weiner A Matcovitch-Natan O Dvir-Szternfeld R Ulland TK. A Unique Microglia Type Associated With Restricting Development of Alzheimer’s Disease. Cell (2017) 169:1276–90.e17. 10.1016/j.cell.2017.05.018 28602351

[35] Heneka MT Kummer MP Stutz A Delekate A Schwartz S Vieira-Saecker A. NLRP3 Is Activated in Alzheimer’s Disease and Contributes to Pathology in APP/PS1 Mice. Nature (2013) 493:674–8. 10.1038/nature11729 PMC3812809 23254930

[36] Fontainhas AM Wang M Liang KJ Chen S Mettu P Damani M. Microglial Morphology and Dynamic Behavior Is Regulated by Ionotropic Glutamatergic and GABAergic Neurotransmission. PloS One (2011) 6:e15973. 10.1371/journal.pone.0015973 21283568 PMC3026789

[37] Liu YU Ying Y Li Y Eyo UB Chen T Zheng J. Neuronal Network Activity Controls Microglial Process Surveillance in Awake Mice *Via* Norepinephrine Signaling. Nat Neurosci (2019) 22:1771–81. 10.1038/s41593-019-0511-3 PMC6858573 31636449

[38] Samardzic T Marinkovic D Danzer C Gerlach J Nitschke L Wirth T. Reduction of Marginal Zone B Cells in CD22-Deficient Mice. Eur J Immunol (2002) 32:561–7. 10.1002/1521-4141(200202)32:2<561::aid-immu561>3.0.co;2-h 11828373

[39] Bolmont T Haiss F Eicke D Radde R Mathis CA Klunk WE. Dynamics of the Microglial/Amyloid Interaction Indicate a Role in Plaque Maintenance. J Neurosci (2008) 28:4283–92. 10.1523/jneurosci.4814-07.2008 PMC3844768 18417708

[40] Zöller T Schneider A Kleimeyer C Masuda T Potru PS Pfeifer D. Silencing of TGFβ Signalling in Microglia Results in Impaired Homeostasis. Nat Commun (2018) 9:4011. 10.1038/s41467-018-06224-y 30275444 PMC6167353

[41] Streit WJ Sammons NW Kuhns AJ Sparks DL. Dystrophic Microglia in the Aging Human Brain. Glia (2004) 45:208–12. 10.1002/glia.10319 14730714

[42] Damani MR Zhao L Fontainhas AM Amaral J Fariss RN Wong WT. Age-Related Alterations in the Dynamic Behavior of Microglia. Aging Cell (2011) 10:263–76. 10.1111/j.1474-9726.2010.00660.x PMC3056927 21108733

[43] Almanzar N Antony J Baghel AS Bakerman I Bansal I Barres BA. A Single-Cell Transcriptomic Atlas Characterizes Ageing Tissues in the Mouse. Nature (2020) 583:590–5. 10.1038/s41586-020-2496-1 PMC8240505 32669714

[44] Naj AC Jun G Beecham GW Wang L-S Vardarajan BN Buros J. Common Variants at MS4A4/MS4A6E, CD2AP, CD33 and EPHA1 Are Associated With Late-Onset Alzheimer’s Disease. Nat Genet (2011) 43:436–41. 10.1038/ng.801 PMC3090745 21460841

[45] Initiative TADN Bradshaw EM Chibnik LB Keenan BT Ottoboni L Raj T. CD33 Alzheimer’s Disease Locus: Altered Monocyte Function and Amyloid Biology. Nat Neurosci (2013) 16:848–50. 10.1038/nn.3435 PMC3703870 23708142

[46] Griciuc A Serrano-Pozo A Parrado AR Lesinski AN Asselin CN Mullin K. Alzheimer’s Disease Risk Gene CD33 Inhibits Microglial Uptake of Amyloid Beta. Neuron (2013) 78:631–43. 10.1016/j.neuron.2013.04.014 PMC3706457 23623698

[47] (ADGC), ADGC, (EADI), TEADI, (CHARGE), C for H and AR in GEC, (GERAD/PERADES), G and ER in AG Polygenic and Environmental Risk for Alzheimer’s Disease Consortium Kunkle BW Grenier-Boley B Sims R Bis JC Damotte V. Genetic Meta-Analysis of Diagnosed Alzheimer’s Disease Identifies New Risk Loci and Implicates Aβ, Tau, Immunity and Lipid Processing. Nat Genet (2019) 51:414–30. 10.1038/s41588-019-0358-2 PMC6463297 30820047

[48] Mrdjen D Pavlovic A Hartmann FJ Schreiner B Utz SG Leung BP. High-Dimensional Single-Cell Mapping of Central Nervous System Immune Cells Reveals Distinct Myeloid Subsets in Health, Aging, and Disease. Immunity (2018) 48:380–95.e6. 10.1016/j.immuni.2018.01.011 29426702

[49] Zhou Y Song WM Andhey PS Swain A Levy T Miller KR. Human and Mouse Single-Nucleus Transcriptomics Reveal TREM2-Dependent and TREM2-independent Cellular Responses in Alzheimer’s Disease. Nat Med (2020) 26:131–42. 10.1038/s41591-019-0695-9 PMC6980793 31932797

[50] Clark EA Giltiay NV. CD22: A Regulator of Innate and Adaptive B Cell Responses and Autoimmunity. Front Immunol (2018) 9:2235. 10.3389/fimmu.2018.02235 30323814 PMC6173129

[51] Linnartz B Wang Y Neumann H. Microglial Immunoreceptor Tyrosine-Based Activation and Inhibition Motif Signaling in Neuroinflammation. Int J Alzheimer’s Dis (2010) 2010:587463. 10.4061/2010/587463 20721346 PMC2915791

[52] Kleinberger G Yamanishi Y Suárez-Calvet M Czirr E Lohmann E Cuyvers E. TREM2 Mutations Implicated in Neurodegeneration Impair Cell Surface Transport and Phagocytosis. Sci Transl Med (2014) 6:243ra86–243ra86. 10.1126/scitranslmed.3009093 24990881

[53] Chong ZZ Maiese K. The Src Homology 2 Domain Tyrosine Phosphatases SHP-1 and SHP-2: Diversified Control of Cell Growth, Inflammation, and Injury. Histol Histopathol (2007) 22:1251–67. 10.14670/hh-22.1251 PMC2515712 17647198

[54] Choi YR Kang S-J Kim J-M Lee S-J Jou I Joe E-H. FcγRIIB Mediates the Inhibitory Effect of Aggregated α-Synuclein on Microglial Phagocytosis. Neurobiol Dis (2015) 83:90–9. 10.1016/j.nbd.2015.08.025 26342897

[55] Zhang L Xia Y Li W Sun Y Kong L Xu P. Activation of Fc Gamma Receptor IIb Up-Regulates the Production of Interferon-Alpha and Interferon-Gamma in Porcine Alveolar Macrophages During PRRSV Infection. Dev Comp Immunol (2020) 109:103696. 10.1007/s13238-017-0473-8 32278861

[56] Wang X Mathieu M Brezski RJ. IgG Fc engineering to modulate antibody effector functions. Protein Cell (2018) 9:63–73. 10.1007/s13238-017-0473-8 28986820 PMC5777978

[57] Taracha A Kotarba G Wilanowski T. Neglected Functions of TFCP2/TFCP2L1/UBP1 Transcription Factors may Offer Valuable Insights Into Their Mechanisms of Action. Int J Mol Sci (2018) 19:2852. 10.3390/ijms19102852 PMC6213935 30241344

[58] Butovsky O Jedrychowski MP Moore CS Cialic R Lanser AJ Gabriely G. Identification of a Unique TGF-β–Dependent Molecular and Functional Signature in Microglia. Nat Neurosci (2014) 17:131–43. 10.1038/nn.3599 PMC4066672 24316888

[59] Spittau B Dokalis N Prinz M. The Role of TGFβ Signaling in Microglia Maturation and Activation. Trends Immunol (2020) 41:836–48. 10.1016/j.it.2020.07.003 32741652

