# Supplementary figures and images for: CD22 Blockage Restores Age-Related Impairments of Microglia Surveillance Capacity
Source: Front Immunol. 2021 Jun 1;12:684430. doi: 10.3389/fimmu.2021.684430 (PMC8204252; doi:10.3389/fimmu.2021.684430)

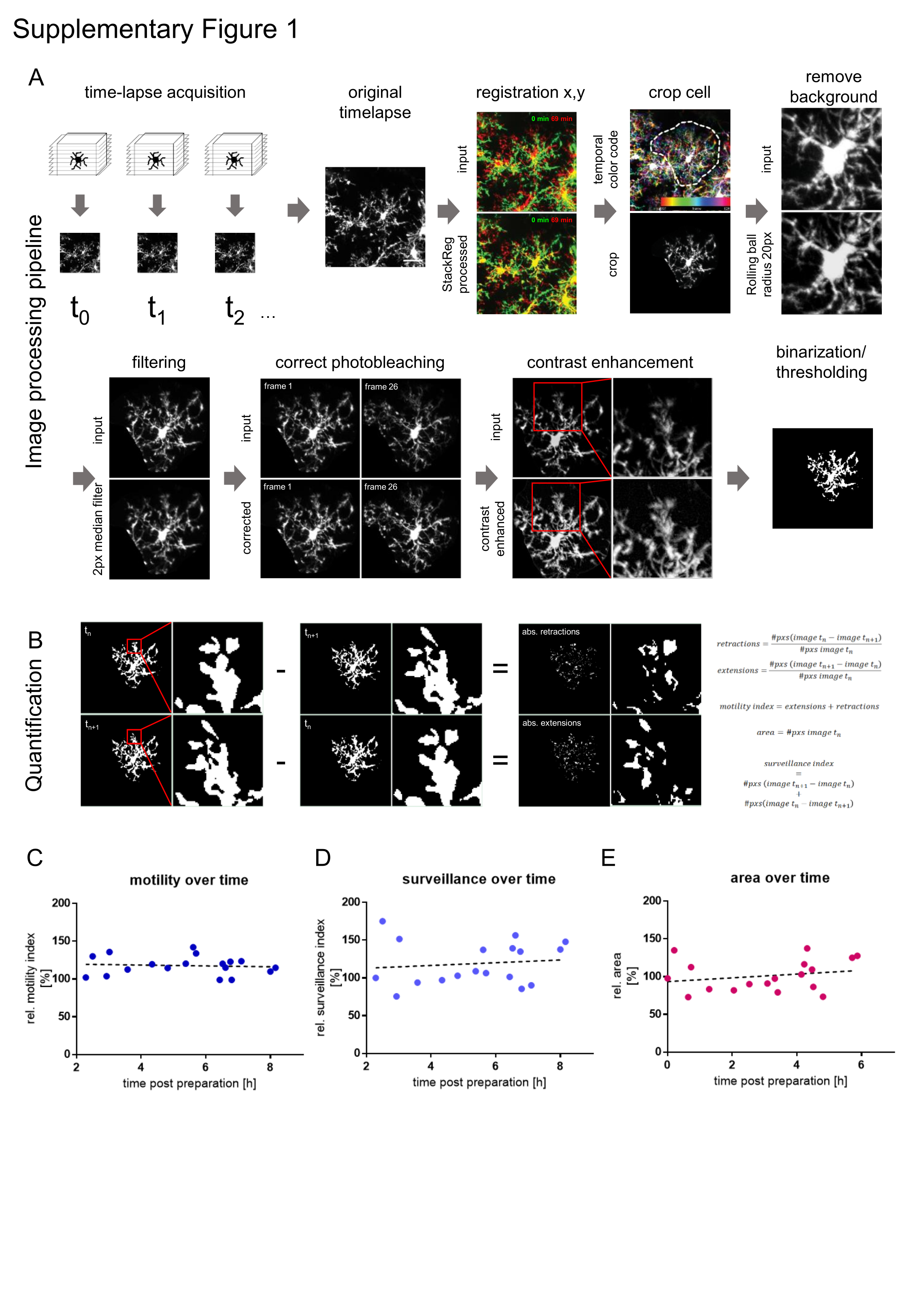

Supplement: Supplementary Figure 1 — Image processing and quantification of two-photon recordings. (A) A maximum intensity projection of the acquired stacks for each timepoint is used to generate the time-lapse. After x,y registration and manual cropping of the cell, an automated series of filters for image correction is applied for thresholding and binarization of the images. (B) To assess the process retractions, image tn is subtracted with tn+1. The results represent the amount of processes in tn which retracted in the following image tn+1. For the process extensions, image tn is subtracted from tn+1, describing the amount of processes which extended from tn to tn+1. While the motility index indicates the microglia process dynamics, the surveillance index serves as a readout for microglial surveillance. Correlation plots of the (C) relative motility, (D) relative surveillance and (E) relative area against the time after slice preparation to evaluate the stability of the measured readouts over time in untreated adult and aged microglia, which were used for experiment in Figure 3. [file Image_1.tif]

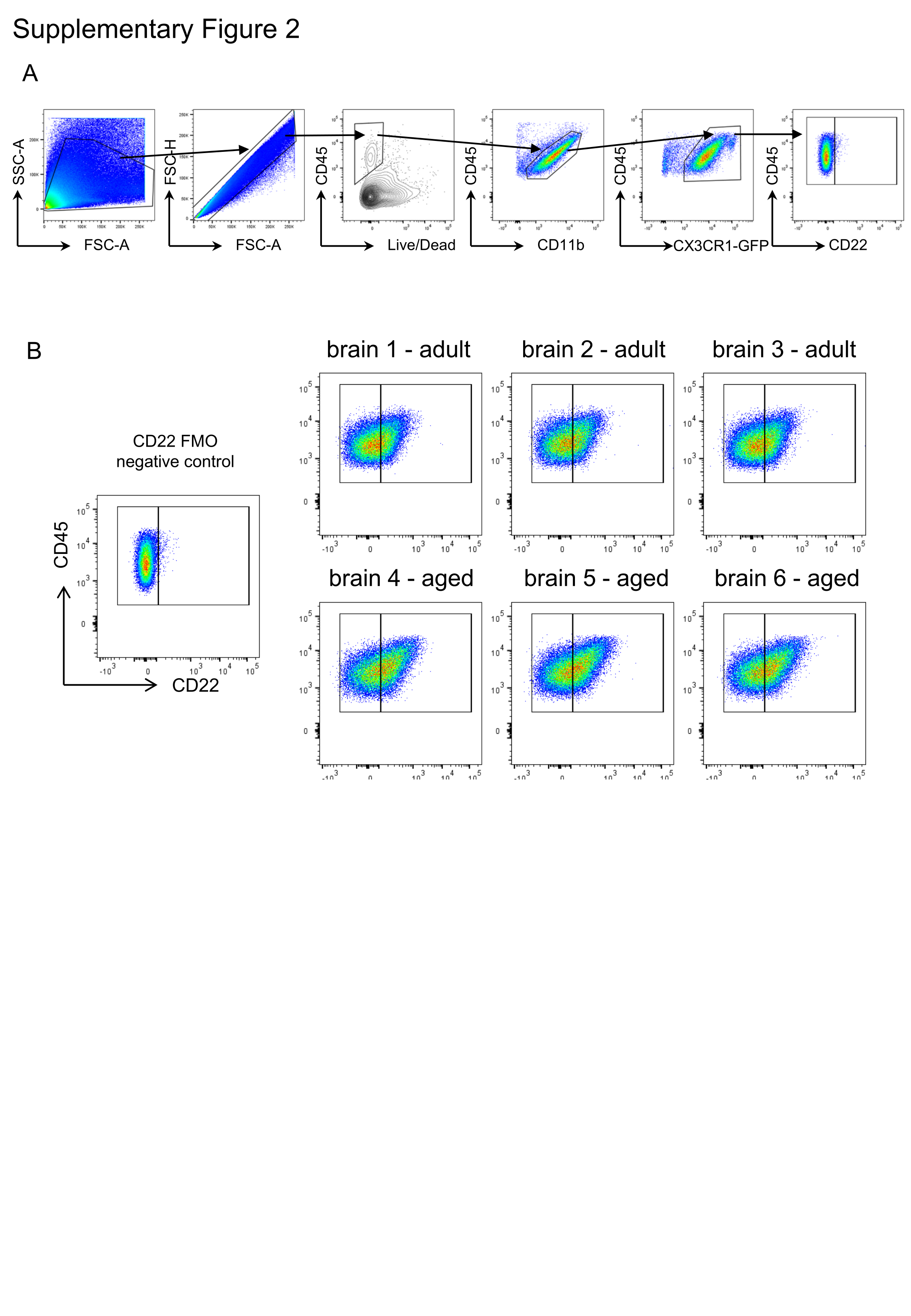

Supplement: Supplementary Figure 2 — Supplementary flow cytometry data. (A) Gating strategy to assess microglial CD22 expression. Cells were gated based on their location on the FCS-A vs SSC-A plot and doublets were excluded consequently (FSC-A vs FCS-H). Live single cells were assessed for CD45 positivity and myeloid cells were gated (CD11b+CD45+ cells). Finally, CD22 expression in CX3CR1 positive myeloid cells (GFP+ cells) was analyzed. (B) Scatter plots to visualize acquired CD22 signal per sample. FMO: Fluorescence Minus One. As control, one sample was stained with all antibodies except the CD22-PE. This sample was used to set the CD22 negative and positive gates, which was then applied on all samples. [file Image_2.tif]

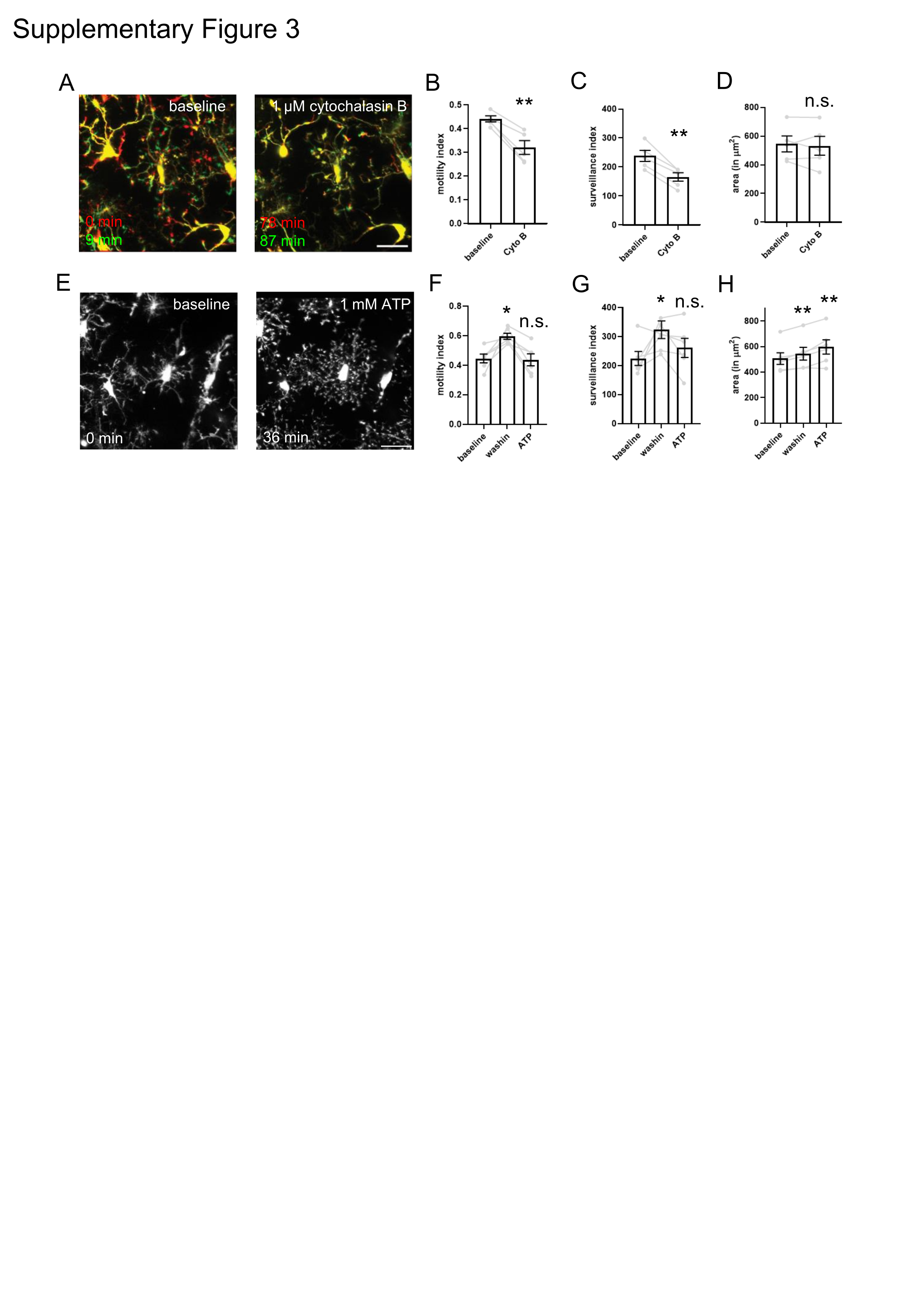

Supplement: Supplementary Figure 3 — Application of other known microglia modulators induce distinct dynamic responses. (A) Wash-in of the actin polymerization inhibitor cytochalasin B diminishes the process speed (B) and the surveilled area (C), while the total area remains stable (D). (E) ATP evokes an increase in size and complexity of the processes and the development of bulbous tips. Upon application of ATP, a reversible increase of motility (E) and surveillance (F) followed by a recovery to steady state, whereas a constant increase of microglia area was measured (G). n=5-6 cells per treatment. Mean ± SEM, (B)-(D) paired t-test, (F)-(H) one-way ANOVA multiple comparisons to baseline, * p<0.05, ** p<0.01, n.s.>0.05. Scale bar: 20 µm. [file Image_3.tif]

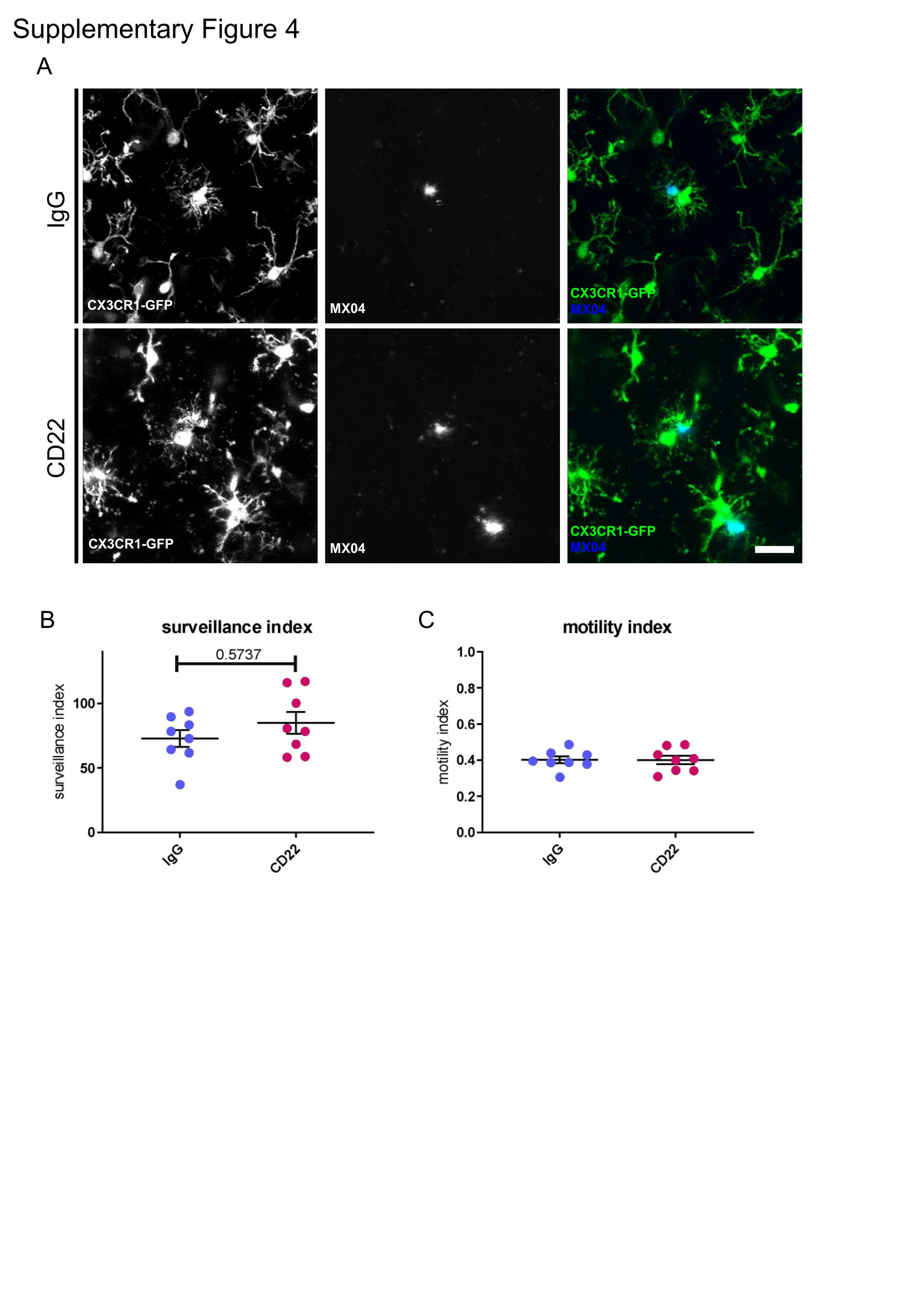

Supplement: Supplementary Figure 4 — Plaque-associated microglia trend towards an increased surveillance capability upon CD22 blockage. (A) Representative two-photon images of plaque-associated microglia in 8-12 months old PS2APP x CX3CR1-GFP mice, which were either treated with a CD22 or an IgG isotype control antibody. Respective surveillance (B) and motility (C) analysis. n=8 cells from n=8 mice, mean ± SEM, unpaired nonparametric t-test (Mann-Whitney), Scale bar: 20 µm. [file Image_4.tif]
